# Racial disparities in HIV pre-exposure prophylaxis (PrEP) awareness and uptake among white, Black, and Indigenous men in Canada: Analysis of data from the *I’m Ready* national HIV self-testing study

**DOI:** 10.17269/s41997-025-01009-5

**Published:** 2025-03-19

**Authors:** Wale Ajiboye, Wangari Tharao, Maureen Owino, Lena Soje, Jason M. Lo Hog Tian, Amy Ly, Margaret Kisikaw Piyesis, Albert McLeod, Mathew Fleury, Kristin McBain, Notisha Massaquoi, Tegan Mosugu, Jaris Swidrovich, Darrell H. S. Tan, LaRon Nelson, Sean B. Rourke

**Affiliations:** 1 MAP Centre for Urban Health Solutions, Unity Health Toronto, Toronto, ON Canada; 2 Women’s Health in Women’s Hands Community Health Centre, Toronto, ON Canada; 3 Faculty of Environmental Studies, York University, Toronto, ON Canada; 4 Women’s College Hospital, Toronto, ON Canada; 5 Canadian Aboriginal AIDS Network, Regina, SK Canada; 6 Faculty of Health Sciences, Simon Fraser University, Burnaby, BC Canada; 7 Department of Health and Society, University of Toronto, Scarborough, ON Canada; 8 Dalla Lana School of Public Health, University of Toronto, Toronto, ON Canada; 9 Faculty of Pharmacy, University of Toronto, Toronto, ON Canada; 10 Temerty Faculty of Medicine, University of Toronto, Toronto, ON Canada; 11 Yale School of Nursing, Yale University, New Haven, CT USA; 12 Department of Psychiatry, University of Toronto, Toronto, ON Canada

**Keywords:** HIV PrEP, Black and Indigenous, PrEP awareness and uptake, PrEP cascade, VIH PrEP, VIH PPrE, Noirs et Autochtones, Sensibilisation à la PrEP, Sensibilisation à la PPrE, Adoption de la PrEP, Adoption de la PPrE, Cheminement PrEP, Cheminement PPrE

## Abstract

**Objectives:**

Black and Indigenous men in Canada continue to experience significant and disproportionate burden of new HIV infection. The purpose of this study was to understand racial differences in PrEP awareness and use, and PrEP cascade among white, Black, and Indigenous men in Canada with the intention to provide evidence for immediate action in our publicly funded health care system.

**Methods:**

We performed a secondary analysis (*n* = 4294) of cross-sectional data from the *I’m Ready* national HIV self-testing research program launched in June 2021 and running through December 2023. Binary logistic regression was used to assess racial differences in PrEP awareness and uptake. A proposed PrEP cascade was developed using the data on awareness, uptake, and retention in PrEP care.

**Results:**

Black participants (OR = 0.34, CI 0.29, 0.39), who are gbMSM (OR = 0.27, CI 0.21, 0.35), aged 18–45 (OR = 0.35, CI 0.30, 0.40), living in urban (OR = 0.41, CI 0.33, 0.51) or rural areas (OR = 0.33, CI 0.26, 0.44), and who are PrEP-eligible (OR = 0.34, CI 0.28, 0.40), were less likely to be aware of PrEP than white participants. Indigenous participants (OR = 0.57, CI 0.44, 0.75), aged 18–45 (OR = 0.57, CI 0.43, 0.75), living in rural communities (OR = 0.15, CI 0.25, 0.57), and who are PrEP-eligible (OR = 0.62, CI 0.46, 0.83), were less likely to be aware of PrEP than white participants. For PrEP uptake, Black participants (OR = 0.61, CI 0.46, 0.82), aged 18–45 (OR = 0.59, CI 0.44, 0.80), living in rural communities (OR = 0.44, CI 0.23, 0.84), and PrEP-eligible (OR = 0.62, CI 0.46, 0.85), were less likely to be on PrEP than white participants. Also, Indigenous men living in urban areas were more likely to be on PrEP than white participants (OR = 1.65, CI 1.01, 2.69).

**Conclusion:**

Community-based and public health interventions are immediately needed to increase PrEP awareness, access, and uptake for Black and Indigenous communities in Canada.

## Résumé

**Objectifs:**

Les hommes noirs et autochtones du Canada continuent d’être victimes d’une surreprésentation importante et même disproportionnée en matière de nouvelles infections au VIH. Le but de cette étude était de comprendre les disparités raciales en ce qui a trait à la sensibilisation et à l’utilisation de la PrEP/PPrE, et plus généralement au cheminement (de l’éligibilité à l’utilisation) relatif à la PrEP/PPrE chez les hommes blancs, noirs et autochtones au Canada. Plus concrètement, notre intention est de fournir des preuves pour qu’une action immédiate soit mise en place au sein de notre système de soins de santé financé par l’état.

**Méthode:**

Nous avons effectué une analyse secondaire (*n* = 4294) des données transversales du programme national de recherche sur l’autodépistage du VIH «I’m Ready/j’agis» lancé de juin 2021 à décembre 2023. La régression logistique binaire a été utilisée pour évaluer les disparités raciales relativement à la sensibilisation et à l’adoption de la PrEP/PPrE. Une proposition de cheminement d’action vis-à-vis de la PrE/PPrE a été élaborée à l’aide des données sur la sensibilisation, l’adoption et la rétention dans les soins de la PrEP/PPrE.

**Résultats:**

Les participants noirs (RC = 0,34, IC: 0,29, 0,39), qui sont des hommes gbHARSAH (RC = 0,27, IC: 0,21, 0,35), âgés de 18 à 45 ans (RC = 0,35, IC: 0,30, 0,40) vivant en milieu urbain (RC = 0,41, IC: 0,33, 0,51) ou en milieu rural (RC = 0,33, IC: 0,26, 0,44), et qui sont éligibles à la PrEP/PPrE (RC = 0,34, IC: 0,28, 0,40), étaient moins susceptibles d’être au courant de la PrEP/PPrE que les participants blancs. Les participants autochtones (RC = 0,57, IC: 0,44, 0,75), âgés de 18 à 45 ans (RC = 0,57, IC: 0,43, 0,75), vivant dans des collectivités rurales (RC = 0,15, IC: 0,25, 0,57) et qui sont éligibles à la PrEP/PPrE (RC = 0,62, IC: 0,46, 0,83), étaient moins susceptibles d’être au courant de la PrEP/PPrE que les participants blancs. En ce qui concerne l’adoption de la PrEP, les participants noirs (RC = 0,61, IC: 0,46, 0,82), âgés de 18 à 45 ans (RC = 0,59, IC: 0,44, 0,80), vivant dans des collectivités rurales (RC = 0,44, IC: 0,23, 0,84) et éligibles à la PrEP/PPrE (RC = 0,62, IC: 0,46, 0,85), étaient moins susceptibles de suivre une PrEP/PPrE que les participants blancs. Par ailleurs, les hommes autochtones vivant en milieu urbain étaient plus susceptibles de suivre une PrEP/PPrE que les participants blancs (RC = 1,65, IC: 1,01, 2,69).

**Conclusion:**

Des interventions communautaires et en santé publique sont immédiatement nécessaires pour accroître la sensibilisation, l’accès et l’adhésion à la PrEP/PPrE dans les communautés noires et autochtones au Canada.

## Introduction

HIV pre-exposure prophylaxis (PrEP) is an effective prevention strategy for reducing the incidence of HIV infection, especially among key populations and vulnerable communities (World Health Organization, 2022). The Government of Canada has identified PrEP as an effective biomedical intervention to achieve zero new infection by 2030, with the specific goal of increasing awareness and access for communities that are impacted by the HIV epidemic (Public Health Agency of Canada, 2024). In the last 5 years, there has been an increase in the proportion of people using PrEP for HIV prevention in Canada (Public Health Agency of Canada 2023b). However, despite the upward trend in PrEP use, many key populations and vulnerable communities in Canada continue to experience an increase in HIV infections (Public Health Agency of Canada, 2023a). Black people in Ontario represent 5.5% of the population, yet they accounted for 25% of new infections in 2020 (Ontario HIV Surveillance Initiative (OHESI), 2022). Furthermore, Indigenous populations represent 5% of the population, but accounted for 18% of new infections in 2020 according to national estimates (Public Health Agency of Canada, 2022). This mismatch between new HIV infections for Black and Indigenous people and the population-level increase in PrEP use is concerning and requires a better understanding of PrEP awareness and uptake in these communities.

Race-based data on PrEP awareness and uptake are currently not available at the provincial or national level (Public Health Agency of Canada, 2023b). There is a lack of work that examines racial differences in PrEP awareness and use, especially among key populations who are most impacted by the HIV epidemic in Canada (Kroch et al., 2023; Ontario HIV Treatment Network (OHTN), 2024; Public Health Agency of Canada, 2023b; Sang et al., 2022). A 2023 Public Health Agency of Canada (PHAC) report on PrEP use in Canada described an increase in PrEP uptake across various provinces without specific details of how the uptake differs among key populations (i.e. people disproportionately affected by HIV in a certain region or country) such as Black populations, Indigenous populations, and gbMSM (gay, bisexual, and other men who have sex with men) (Public Health Agency of Canada, 2023b). A study by Sang et al. (2022) examined provincial PrEP coverage and characteristics of PrEP awareness and use among gbMSM in Vancouver, Toronto, and Montreal from 2017 to 2020, highlighting the significance of publicly funded PrEP coverage and the impact on PrEP awareness and use. However, this study only examined PrEP use among one key population (gbMSM) across three cities. There are currently no national estimates of racial differences in PrEP awareness and PrEP use and its associated characteristics across different parts of the country, especially among cis-men, who bear a great burden of new HIV infection in Canada. Understanding these differences is required to address racial inequities associated with PrEP as well as HIV.

Evidence suggests that PrEP implementation in key populations can lead to significant drops in HIV infection. An increase in PrEP uptake and a nurse-led PrEP intervention were reported to coincide with a decrease in new infections among gbMSM in Ottawa (Kroch et al., 2023). Furthermore, some studies have also shown that without PrEP implementation, racial disparities in HIV infection will continue to increase. For example, Goedel et al. (2020) predicted that the HIV incidence rate for Black/African American MSM was 3.82 times greater than that for white MSM in the absence of PrEP implementation.

To realize the public health benefits of PrEP, implementation must be targeted to populations with the highest risk for acquiring HIV and for those with indications for PrEP. Recent national epidemiological data show that male-to-male contact accounts for over half of new infections in 2022 (Public Health Agency of Canada, 2023a). In Ontario, one out of every four new HIV diagnoses in 2022 are from Black communities (Ontario HIV Surveillance Initiative (OHESI), 2022). In 2020, almost 70% of new HIV infections were among males in Indigenous communities (Public Health Agency of Canada, 2022). Finally, gbMSM continue to bear a disproportionate burden of HIV in Canada, with 166 new infections per 100,000 population compared to 4 new infections per 100,000 Canadians in the general public (Public Health Agency of Canada, 2022). In terms of PrEP indication, the Canadian guideline on HIV pre-exposure prophylaxis and nonoccupational postexposure prophylaxis (PEP) strongly recommends PrEP for gbMSM who are sexually active and have ongoing risk for acquiring HIV (Tan et al., 2017). Therefore, for this analysis, we sought to (1) understand racial differences and factors associated with PrEP awareness and uptake among white, Black, and Indigenous cis-male; and (2) estimate and describe racial differences in a proposed PrEP awareness and uptake cascade. A PrEP cascade shows the stages of engagement with PrEP as an HIV prevention strategy. Different variables have been used to describe PrEP cascade but the most common variables include PrEP awareness (being aware of PrEP as a tool to prevent HIV infection); willingness to use PrEP (self-motivation to initiate and adhere to PrEP); PrEP use (uptake and use of PrEP); and PrEP adherence (persistence or using PrEP for as long as needed) (Mistler et al., 2021).

## Methods

### Study design

We conducted a secondary analysis of cross-sectional data derived from the *I’m Ready* national HIV self-testing research program. This study analyzed PrEP data for white, Black, and Indigenous men to identify and describe racial differences in awareness and uptake, and the characteristics associated with such differences. We also estimated a PrEP cascade (using data available) for the three racial groups.

### About the *I’m Ready* HIV self-testing research program

*I’m Ready* is the first Canadian national HIV self-testing research program that uses a mobile app to distribute free HIV self-test kits to Canadians, either through delivery to an address of choice or through pick-up from a local community organization. Participants can download the mobile app via the Apple App Store (iOS users) or Google Play Store (Android users). Before ordering self-test kits, participants answered a pre-test survey about sociodemographic characteristics, recent testing, sexual risk behaviour, and PrEP awareness and use.

Enrollment in the study required participants to be Canadian residents, capable of providing online consent in English or French, and at least 18 years of age, and to have either negative or unknown HIV status, and have a cellular phone. Exclusion criteria included being aware of one’s HIV-positive status. Prospective participants were recruited through multiple channels, including community referral (REB-approved advertisements, word of mouth) and public campaigns (including social media and project websites).

All data collected from the app were stored on private servers in Canada with the Amazon Web Services (AWS) secure cloud. Once the data were downloaded and retrieved from AWS, they were saved and stored on Unity Health Toronto servers. Data were password protected, regularly backed up, and only accessed by approved team members at Unity Health Toronto. This study was approved by the Research Ethics Board at Unity Health Toronto (REB#: 20–283). This study uses cross-sectional data from the pre-test survey collected nationwide between June 2021 and December 2023.

### Measures

#### PrEP awareness and uptake

To assess awareness of PrEP, participants were asked, “Have you ever heard of HIV Pre-Exposure Prophylaxis or PrEP, an HIV medication taken routinely by HIV-negative people to help prevent HIV infection?”, with options to answer yes or no. To evaluate the uptake of PrEP, participants were asked “Have you ever used HIV Pre-Exposure Prophylaxis (PrEP)” and could respond with “No, I have never taken PrEP”, “Yes, but I stopped and have not used it since”, or “Yes, I am taking PrEP now!” Both “unsure” and “prefer not to answer” options were provided for each question, allowing participants to opt out of answering if they felt uncomfortable. For analyses, awareness and uptake of PrEP were dichotomized as “yes” versus “no”. People who answered “unsure” or “prefer not to answer” were excluded from analyses.

#### PEP uptake

For PEP uptake, participants were asked, “Have you ever received Post-Exposure Prophylaxis, a 4-week course of medications that you can take if you are HIV negative and think you have been recently exposed?”, with options to answer yes or no. Participants were also able to answer “unsure” or “prefer not to answer”. These participants were excluded from analyses.

#### Sexual behaviour risk

A sexual risk behaviour score was calculated based on two survey questions asking about condomless anal or vaginal sex in the past 3 months and the number of sexual partners of unknown HIV status in the last 12 months. Participants who did not have condomless sex in the past 3 months and no condomless sex with partners of unknown HIV status in the past 12 months had “low” sexual risk behaviour indicators. Participants who had condomless sex in the past 3 months and/or condomless sex with at least one partner of unknown HIV status in the past 12 months had “high” sexual risk behaviour indicators. Participants who answered yes to “Have you ever injected illicit drugs?” were also classified as having “high” risk behaviour indicators.

### Statistical analysis

Participant characteristics and key variables of interest were summarized using descriptive statistics, including counts and percentages. We estimated a PrEP cascade for those eligible for PrEP by calculating the percentage of participants with high sexual risk behaviour who are aware of PrEP, received PrEP, and were retained on PrEP. Binary logistic regression was used to assess whether race was associated with having heard of PrEP and having received PrEP with people identifying as white acting as the reference group. We used white as reference group because it is the major racial group in the sample. To understand racial differences in different epidemiological groups, the logistic regression was then stratified by those who identified as gay, bisexual, and other men who have sex with men (gbMSM), those under 45 years old, those who lived in urban areas (population greater than or equal to 100,000 people), those who lived in rural areas (population less than 100,000 people) (Statistics Canada, 2021), those who received post-exposure prophylaxis (PEP), and those who had high sexual behaviour risk. Odds ratios, their 95% confidence intervals, and *p*-values were reported. A *p*-value less than 0.05 was considered statistically significant. Variables were selected based on population characteristics that are associated with PrEP use. Variables were all entered into models simultaneously. Hosmer–Lemeshow tests were used to assess model fit, with a *p*-value greater than 0.05 indicating a good fit. Participants without data points for a particular variable were removed using pairwise deletion, with each antecedent variable and outcome serving as the pairs. All analyses were conducted using IBM SPSS Statistics version 24.

## Results

### Study participants

Table 1 describes the participant demographics (*n* = 4294), including age, gender, sexual orientation, race, sexual risk behaviour, PEP use, and PrEP awareness and use. Participants were mostly under 45 years old (90%). For race, participants identified as white (56%), Black (36%), or Indigenous (8%). For sexual orientation, most people identified as either straight (28%) or gay (39%) and over half identified as gbMSM (57%). Most participants were from very large urban areas (47%) and were identified as having high sexual behaviour indicators (85%).

**Table 1:** Participant demographics and variables of interest (*n* = 4294)

	*n*	Percent
Age		
18–24	1329	31%
25–34	1668	39%
35–44	878	20%
45–54	276	6%
55 +	143	3%
Race		
White	2415	56%
Black	1548	36%
Indigenous	331	8%
Sexual orientation		
Straight	1124	28%
Gay	1548	39%
Bisexual	478	12%
Other*	816	21%
gbMSM		
gbMSM	2434	57%
Non-gbMSM	1860	43%
Location size		
Very large urban (≥ 200,000)	1803	47%
Large urban (100,000–199,999)	584	15%
Medium (30,000–99,999)	501	13%
Small (≤29,999)	936	25%
Sexual behaviour risk		
Low risk	521	15%
High risk	3015	85%
Received PEP		
Yes	291	8%
No	3217	90%
Unsure	78	2%
Heard of PrEP		
Yes	2232	62%
No	1285	36%
Unsure	69	2%
Received PrEP		
Yes, currently taking	159	7%
Yes, but stopped	262	12%
No	1766	80%
Unsure	20	1%

*Other sexual orientations include queer, pansexual, heteroflexible, asexual, and questioning

Only 291 (8%) participants had ever received PEP. Among those who answered questions on PrEP awareness and use, 2232 (62%) were aware of PrEP, 421 (19%) had received PrEP, and 262 (12%) participants who received PrEP had stopped using the medication.

### Characteristics associated with PrEP awareness

Table 2. shows PrEP awareness and uptake among different racial identities as well as racial identities stratified by those who are gbMSM, under 45 years old, living in urban areas (≥ 100,000) and rural areas (< 100,000), who have used PEP, and with high sexual behaviour indicators.

**Table 2.** Logistic regression with variables of interest predicting awareness and uptake of PEP and PrEP

	Heard of PrEP			Received PrEP		
	OR	95% CI	*p*	OR	95% CI	*p*
Race						
White		ref			ref	
Black	0.34	(0.29, 0.39)	** < **0.01	0.61	(0.46, 0.82)	** < **0.01
Indigenous	0.57	(0.44, 0.75)	** < **0.01	1.42	(0.95, 2.13)	0.09
Race + gbMSM						
White gbMSM		ref			ref	
Black gbMSM	0.27	(0.21, 0.35)	** < **0.01	1.03	(0.71, 1.51)	0.87
Indigenous gbMSM	0.73	(0.49, 1.08)	0.12	1.42	(0.92, 2.17)	0.11
Race + age						
White < 45		ref			ref	
Black < 45	0.35	(0.30, 0.40)	** < **0.01	0.59	(0.44, 0.80)	** < **0.01
Indigenous < 45	0.57	(0.43, 0.75)	** <** 0.01	1.25	(0.81, 1.91)	0.31
Race + urban (≥ 100,000)						
White urban		ref			ref	
Black urban	0.41	(0.33, 0.51)	** <** 0.01	0.69	(0.47, 1.00)	0.05
Indigenous urban	1.03	(0.68, 1.57)	0.90	1.65	(1.01, 2.69)	0.05
Race + rural (< 100,000)						
White rural		ref			ref	
Black rural	0.33	(0.26, 0.44)	** <** 0.01	0.44	(0.23, 0.84)	0.01
Indigenous rural	0.38	(0.25, 0.57)	** < **0.01	1.15	(0.51, 2.58)	0.74
Race + PEP use						
White and used PEP		ref			ref	
Black and used PEP	0.10	(0.04, 0.28)	** <** 0.01	0.51	(0.29, 0.90)	0.02
Indigenous and used PEP	0.15	(0.04, 0.62)	0.01	0.30	(0.11, 0.88)	0.03
Race + risk						
White ‘high risk’		ref			ref	
Black ‘high risk’	0.34	(0.28, 0.40)	** < **0.01	0.62	(0.46, 0.85)	** < **0.01
Indigenous ‘high risk’	0.62	(0.46, 0.83)	** < **0.01	1.34	(0.88, 2.06)	0.17

Those identifying as Black (OR = 0.34, CI 0.29, 0.39) or Indigenous (OR = 0.57, CI 0.44, 0.75) were less likely to have heard of PrEP than those identifying as white. Among gbMSM, those identifying as Black gbMSM (OR = 0.27, CI 0.21, 0.35) were less likely to have heard of PrEP than those identifying as white gbMSM. For those under 45 years of age, Black (OR = 0.35, CI 0.30, 0.40) and Indigenous (OR = 0.57, CI 0.43, 0.75) individuals were less likely to have heard of PrEP. Among those living in an urban area, people identifying as Black (OR = 0.41, CI 0.33, 0.51) were less likely to have heard of PrEP. Among those living in a rural area, Black (OR = 0.33, CI 0.26, 0.44) and Indigenous (OR = 0.15, CI 0.25, 0.57) were less likely to have heard of PrEP. For those who had an indication for PrEP based on previous use of PEP, Black (OR = 0.10, CI 0.04, 0.28) and Indigenous (OR = 0.15, CI 0.04, 0.62) were all less likely to have heard of PrEP. Among those who had indications for PrEP based on their sexual risk behaviour, Black (OR = 0.34, CI 0.28, 0.40) and Indigenous (OR = 0.62, CI 0.46, 0.83) individuals were less likely to have heard of PrEP.

### Characteristics associated with PrEP uptake

For uptake of PrEP, those identifying as Black were less likely to have received PrEP than those identifying as white (OR = 0.61, *p* < 0.01). Those identifying as Black and under 45 years old (OR = 0.59, *p* < 0.01) were less likely to have received PrEP than those identifying as white. Among those living in an urban area (≥ 100,000), people identifying as Indigenous (OR = 1.65, *p* = 0.05) were more likely to have received PrEP than those identifying as white. Among those living in a rural area (< 100,000), those identifying as Black (OR = 0.44, *p* < 0.01) were less likely to have heard of PrEP. For those who had indication for PrEP based on previous use of PEP, Black (OR = 0.51, *p* = 0.02) and Indigenous (OR = 0.30, *p* = 0.03) were less likely to have received PrEP. Among those with indications for PrEP based on their sexual risk behaviour, those identifying as Black were less likely to have received PrEP (OR = 0.62, *p* < 0.01). Hosmer–Lemeshow tests showed a good fit for all logistic regression models.

### PrEP cascade by race

**Fig. 1:**
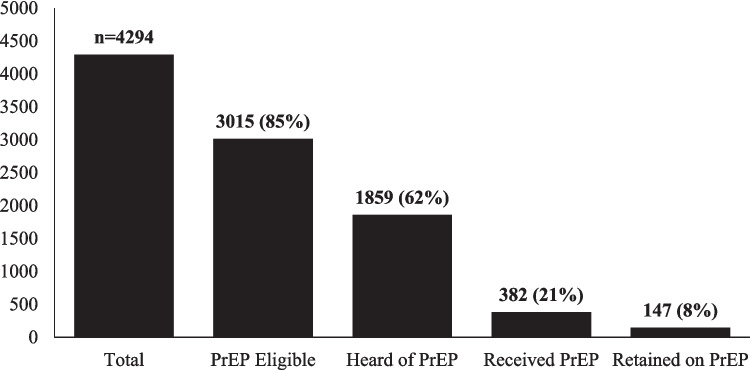
PrEP cascade

**Fig. 2:**
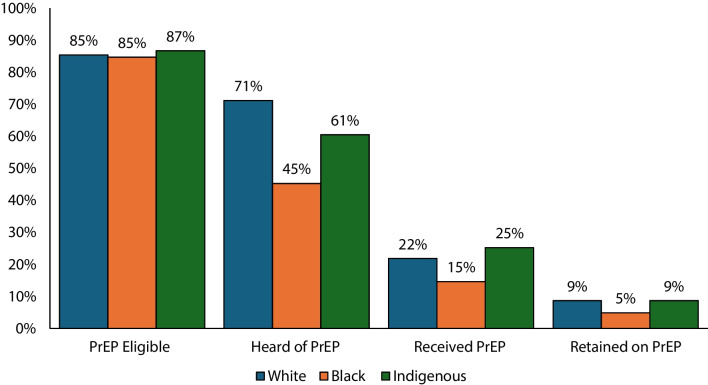
PrEP cascade by race

Table 3 shows a proposed PrEP cascade by race. Overall, 62% of those eligible for PrEP were aware of PrEP, 21% received PrEP, and 8% were currently using PrEP (Fig. 1). When comparing the PrEP cascade among different racial identities (Fig. 2), participants identifying as Black had the lowest percentages of those who were aware of PrEP (45%), received PrEP (15%), and retained PrEP (5%).

**Table 3:** PrEP cascade by race

	Total		PrEP eligible		Heard of PrEP		Received PrEP		Retained on PrEP	
	*n*	Percent	*n*	Percent	*n*	Percent	*n*	Percent	*n*	Percent
Total	4294	100%	3015	85%	1859	62%	382	21%	147	8%
White	2415	100%	1900	85%	1343	71%	293	22%	117	9%
Black	1548	100%	894	85%	389	45%	57	15%	19	5%
Indigenous	331	100%	221	87%	127	61%	32	25%	11	9%

## Discussion

### Summary of results and implications

Race-based data are not collected as part of routine care in Canada, so it is challenging to ascertain PrEP access and use among different racial identities. Our study is the first national study to identify and describe characteristics associated with racial differences in PrEP awareness and uptake among cis-men. Our results show that Black and Indigenous cis-males who identify as gbMSM, aged 18–45 years, and who are PrEP eligible are less likely to be aware of or receive PrEP than their white counterparts. This result highlights disparities in PrEP awareness and use in communities that are most impacted by the HIV epidemic in Canada. This mismatch between PrEP need and PrEP awareness/use for communities with the highest risk for acquiring HIV and for people who have an indication for PrEP use is deeply concerning and the implication could be far reaching. Canada may not be able to reach the goal of zero new infections by year 2030 if the PrEP racial disparities for these communities are not addressed. Although no other study has compared differences in PrEP use among different racial identities in Canada, several studies in the United States and United Kingdom that examined these differences have reported similar results. Sullivan et al. (2024) examined equity of PrEP uptake by race, ethnicity, gender, and region, and concluded that PrEP equity for Black and Hispanic people decreased from 2012 to 2021. Other similar studies on trends in PrEP awareness, willingness, and use have also reported a mismatch between PrEP use and epidemic need, especially for Black and Hispanic gbMSM (Bunting et al., 2023; Coukan et al., 2024; Kanny et al., 2019; Schexnayder et al., 2022; Sullivan et al., 2020).

A PrEP cascade can provide measurable indicators to track progress along the prevention framework, from the stage of identifying individuals who are PrEP eligible to the stage of persistence in PrEP care (Brouwers, 2015). Our study shows a progressive reduction in the proportion of people retained along the PrEP cascade, from those who are eligible, to those who are aware of PrEP, to those who received PrEP, and to those who are retained on PrEP. Although the pattern was similar among the various racial groups, people identifying as Black had the highest proportion of people dropping off the PrEP cascade. Our result may explain the reason new infection rates in these communities remain the same despite increase in reported national PrEP uptake from 2019 to 2022 (Public Health Agency of Canada, 2023b). Second, our result suggests that PrEP programs at national or provincial levels are not having the expected impact among Black and Indigenous communities because the percentage of PrEP-eligible individuals who are retained on the PrEP cascade is a key measure of the success of any PrEP implementation program (World Health Organization, 2022). In summary, our results highlight two important areas for discussion. First, this result provides a basis to highlight the context for the observed PrEP racial disparities which have been previously reported but have not gained the expected attention due to lack of data on the impact of these factors. Second, our result provides evidence-for-action for policy makers, providers, and researchers to develop and implement transformative actions required to reduce racial PrEP disparities in Canada.

### Context for the observed racial PrEP disparities

For several years, studies have highlighted the significance of structural barriers (anti-Black and anti-Indigenous racism, systemic marginalization) and social determinants of health as factors affecting Black and Indigenous people’s ability to access and be retained in care including HIV prevention services. Although our study did not seek to establish a causal relationship, the characteristics associated with PrEP awareness and use, and the racial differences in PrEP cascade suggest that structural barriers and social determinants of health may be responsible for the observed PrEP racial disparities among Black and Indigenous communities. For example, previous studies have observed the link between racial identity and provision of PrEP information. A recent Gay Men’s Sexual Health Alliance (GMSH) Black Same Gender Loving Men community report highlighted that anti-Black racism in the health care system drove the selective exclusion of PrEP information from Black patients; physicians were reluctant to discuss or prescribe PrEP, and Black men were hesitant to accept PrEP due to mistrust (Absalom & Boyce, 2020). Also, Ajiboye et al. (2022) identified lack of adequate knowledge or insufficient knowledge, especially gaps in information provided by healthcare providers, as some of the reasons PrEP-eligible Black patients rejected PrEP for HIV prevention. Similarly, social determinants of health like poverty, education, employment, and immigration status have also been linked to factors affecting access to HIV prevention services. More specifically, among heterosexual Black men in London, Ontario, full-time employment was significantly associated with higher odds of HIV testing in this population (Konkor et al., 2020). This is significant given that HIV testing is the entry point of the PrEP care continuum. Also, in the 2019 Trans PULSE Canada survey of transgender and nonbinary individuals in Canada on PrEP awareness and use, respondents who were Indigenous, living in Atlantic Canada or Quebec, and having high school or less were significantly less likely to be aware of PrEP (Hallarn et al., 2022). To understand and correctly interpret the reasons for the observed PrEP racial disparities among Black and Indigenous communities in Canada, our results must be interpreted from the lens of these systemic and structural barriers which make it difficult for these communities to access and benefit from effective health interventions.

### Recommendations for transformative actions required to reduce racial PrEP disparities

To reduce the racial PrEP disparities in Canada, transformative actions are needed at national, provincial, and local community levels to initiate, implement, and scale up policies, programs, and practices that will address the following areas:

1. Use a decolonizing framework to develop and implement PrEP policies, programs, and practices: This will help to address the structural and systemic barriers that disadvantaged Black and Indigenous communities from accessing and utilizing PrEP for HIV prevention. Policy makers, program implementers, and providers should consider the use of decolonizing frameworks, especially those that address anti-Black and anti-Indigenous racism, when formulating PrEP policy, programs, and practice.
2. Develop a national PrEP strategy: A national government initiative is needed to promote the awareness and use of PrEP for HIV prevention. To be effective, the Government of Canada must use an equity lens to formulate and implement a PrEP national strategy—specifically, an equity lens for addressing PrEP care that is grounded in free access to PrEP for individuals who often face economic challenges to accessing and sustainably using PrEP. Based on the findings of this study, Black and Indigenous communities need to be prioritized for allocation of resources and involvement in the development of a PrEP national strategy. There may be considerable public health benefits to increasing PrEP equity for these communities as several models have shown a decrease in HIV infection rate if the proportion of PrEP use relative to PrEP need (PrEP-to-need-ratio) is increased (Goedel et al., 2020).
3. Collect and use race-based data to guide HIV prevention research, program, and policy agenda: Results from our study emphasize the importance of collecting race-based data in routine clinical and public health services. Without race-based data, it is difficult to achieve equitable access to effective and much-needed interventions to help communities. Race-based data will provide evidence for targeted intervention and efficient use of resources, accelerating progress towards achieving public health goals. Our study is one of the first to offer national population-level insight into racial differences in PrEP awareness and uptake, and to estimate a PrEP cascade for Black and Indigenous peoples in Canada. Disaggregated prescribing data that include information on racial, gender, and sexual identities, as well as location, are needed to monitor the progress of a national PrEP program. Without these data, it will be challenging to ensure equitable access to PrEP, reduce HIV disparities, or achieve the vision of zero new infections by 2030.
4. Implement effective interventions: Campaigns to promote PrEP awareness, willingness to use, and uptake, particularly in Black and Indigenous communities in Canada, are urgently needed. These campaigns are critical in creating more social awareness, therefore boosting demand generation and increasing uptake. In order to be successful, they must be community-driven and culturally appropriate and must reflect community priorities and preferences. These campaigns should utilize various forms of traditional and digital media, such as social media apps, dating apps, and television. Additionally, they should engage and prioritize various community members, thus creating a more collaborative process for formulating key messages to the public and incorporating the perspectives of community members with lived/living experience, service providers, and leaders in the communities throughout the process. We believe our results also highlight the significance of using multi-component interventions to address the issue of PrEP access, uptake, and retention in care for Black and Indigenous communities. Multi-level system approach and multi-component solutions that target structural, social, behavioural, and biomedical determinants of PrEP acceptance, access, uptake, and retention should be prioritized above individual-level interventions if these communities are to have equitable access to PrEP’s public health benefits for HIV prevention. For example, evidence suggests that models with interventions that target multiple points in the PrEP cascade, such as awareness, willingness to use, linkage to care, and adherence, have been more successful among Black sexual minority men in increasing PrEP uptake and use compared to those that target a single point in the cascade (Turpin et al., 2022).

### Limitations

We caution that our results should be interpreted in light of some limitations. This is a cross-sectional analysis of population-level data with data from a specific time point, which limits our ability to draw conclusions on how PrEP awareness and uptake have changed over time. Participants were free to decline to answer any survey questions so as not to prevent anyone getting access to HIV self-testing; however, this creates gaps in the data which may cause response bias. Sexual behaviour risk was assessed using two questions; however, this is an imperfect and incomplete picture of the factors that comprise PrEP eligibility. Future work must assess whether the findings from this work persist in the context of a more comprehensive PrEP eligibility score. Moreover, the non-availability of other national data on PrEP (e.g. prescribing data disaggregated by race/ethnicity, location, and gender) limits our ability to see how our results compare with other epidemiological data. The lack of data on “willingness to use PrEP” is another limitation and should be assessed alongside awareness and uptake. Data on willingness to use PrEP would provide important insight into those who are willing to use it but unable to access it. Our estimate of the PrEP cascade is based on the data available from this research program; however, it may not accurately reflect the actual cascade since this is not a cohort study of participants prescribed PrEP. It does, however, provide insight into the level of PrEP use, and the discontinuation of PrEP among those who had initially agreed to use it. Further research should explore PrEP acceptance/willingness to use, affordability, and amount of time since starting PrEP before discontinuation; all these factors could help inform a more robust determination of the PrEP cascade. In addition, further research could also focus on determining the PrEP-to-need ratio and PrEP-equity ratio for Black and Indigenous communities. These PrEP use indicators will enable policy makers to monitor progress on increasing PrEP equity for these communities.

## Conclusion

Black and Indigenous cis-men in Canada experience racial differences in PrEP awareness, uptake, and adherence in comparison with their white counterparts. To maximize the potential benefits of PrEP for HIV prevention, transformative actions that address anti-Black, anti-Indigenous racism and social determinants of health, and a national PrEP strategy that is based on equity are needed urgently. Black and Indigenous cis-men identifying as gbMSM, aged 18–45 years, and living in rural communities should be prioritized and targeted for PrEP awareness campaigns and PrEP services to increase PrEP uptake and use. Given that there are limited facility-based services in rural communities, the use of technology to increase PrEP awareness, facilitate access to PrEP providers, and enhance delivery of PrEP through mail or easily accessible community centres may be particularly useful in these communities. In addition, for PrEP awareness to be effective, it should be community-driven and culturally appropriate and must target at-risk groups (cis-men, gbMSM) within these communities. Further research may be needed to understand the barriers to PrEP awareness, acceptance, and retention in care in order to fully develop and implement interventions that will be effective in changing the PrEP cascade for these communities. Finally, all stakeholders in the HIV ecosystem—public health units, policymakers, funders, service and clinical providers, researchers, and people with lived experience—should prioritize and develop action plans to increase PrEP awareness and use in these communities in order for Canada to meet the target of zero new infections by 2030.

## Contributions to knowledge

What does this study add to existing knowledge?

- This study advanced the knowledge of PrEP inequities for two key populations (Black and Indigenous communities) that are disproportionately impacted by the HIV epidemic in Canada.

What are key implications for public health interventions, practice, and policy?

- Evidence from this study will enhance the design and implementation of targeted public health interventions to increase PrEP awareness and uptake for Black and Indigenous communities in Canada.

## Data Availability

N/A.

## Code availability

N/A.
